# Quantification of Ash and Moisture in Wheat Flour by Raman Spectroscopy

**DOI:** 10.3390/foods9030280

**Published:** 2020-03-03

**Authors:** Tomasz Czaja, Aldona Sobota, Roman Szostak

**Affiliations:** 1 Department of Chemistry, University of Wrocław, 14F, Joliot-Curie, 50-383 Wrocław, Poland; tomasz.czaja@chem.uni.wroc.pl; 2 Department of Food Science, University of Copenhagen, Rolighedsvej 26, 1958 Frederiksberg, Denmark; 3 Engineering and Cereals Technology Department, University of Life Sciences in Lublin, Skromna 8, 20-704 Lublin, Poland; aldona.sobota@up.lublin.pl

**Keywords:** wheat flour, ash, moisture, protein, elemental analysis, multivariate analysis

## Abstract

Wheat flour is widely used on an industrial scale in baked goods, pasta, food concentrates, and confectionaries. Ash content and moisture can serve as important indicators of the wheat flour’s quality and use, but the routinely applied assessment methods are laborious. Partial least squares regression models, obtained using Raman spectra of flour samples and the results of reference gravimetric analysis, allow for fast and reliable determination of ash and moisture in wheat flour, with relative standard errors of prediction of the order of 2%. Analogous calibration models that enable quantification of carbon, oxygen, sulfur, and nitrogen, and hence protein, in the analyzed flours, with relative standard errors of prediction equal to 0.1, 0.3, 3.3, and 1.4%, respectively, were built combining the results of elemental analysis and Raman spectra.

## 1. Introduction

Wheat flour is the most important product of wheat milling. It is used on an industrial scale in baking and in producing confectionaries, pasta and food concentrate. Ash is one of the major indicators of wheat flour’s quality and use [1,2]. The ash obtained from flours consists of mineral compounds of phosphorous, potassium, calcium, magnesium, iron, zinc, and copper. Phosphorus (approximately 45%), potassium (approximately 38%), magnesium, and calcium (approximately 13% and 3%, respectively) are the main elements present in ash, while the other elements amount to only 1% [3,4]. The whole wheat grain contains 1.17–2.96% of the mineral constituents [5]. This variation is caused by the genotype, wheat class and cultivar as well as the growing location and year [4]. Minerals in the kernel are distributed unevenly. The aleurone layer and pericarp contain approximately 68%, the starch endosperm 20%, and the embryo 12% of the total minerals [6]. Flour characterized by a higher ash level is usually less purified and contains more particles of fine bran and endosperm adjacent to the bran. Therefore, ash is a widely used index of flour purity and its extraction rate during milling [4]. However, it should be noted that some wheat types, e.g., durum wheat, naturally have a higher level of endosperm ash due to genetic factors and soil conditions. From a nutritional point of view, an increase in the ash content in flour combined with an increase in the content of dietary fiber, vitamins, and non-gluten proteins is desirable [7]. However, the technical quality of high-ash flour is lower because it is characterized by a darker color and greater activity of proteolytic and amylolytic enzymes. Dietary fiber and non-gluten proteins disintegrate and weaken the protein matrix during dough formation [2,8]. Therefore, the ash content in flour is an important parameter in the assessment of flour quality. Measurement of the ash content is routinely performed using a standard ash analysis method in which the sample is burned at 550 °C for soft wheat flours and 575–590 °C for hard wheat flours. Incinerating is carried out until light gray ash is obtained or until a constant weight is reached. The time of this determination is long and varies from 5 to 7 h. In an industrial practice, this method is not frequently used because the time needed is too long and does not allow the wheat flour’s quality to be verified effectively [9,10]. Numerous instrumental techniques have been proposed for ash and moisture analysis in different types of flour samples. Undoubtedly the most important and often applied in an industrial practice, is near-infrared spectroscopy (NIR) [11]. Other techniques include ATR (attenuated total reflection), infrared transmission and laser-induced breakdown spectroscopy [9,12,13,14] 

In this report, we present a new, fast, and reliable Raman spectroscopic method for ash, moisture, and protein quantification in wheat milling fractions. Portable Raman spectrometers, widely available now, can be used to adapt this type of analysis in production facilities. Raman spectroscopy is a versatile analytical method that delivers unique information about molecules on the basis of their oscillations. It enables qualitative and quantitative analysis of different compounds present in the studied samples, and it is much more specific than NIR spectroscopy [15].

## 2. Materials and Methods 

### 2.1. Milling Process

Flours obtained from milling 15 different cultivars of common wheat (Triticum aestivum) were used. Wheat grain (ten spring wheat genotypes cv. Kamelia, Katoda, Monsun, Narwa, Ostka Smolicka, Raweta, Goplana, Tybalt, Fala, Kandela and five winter genotyps cv. Bogatka, Smuga, Pokusa, Tonacja, Skagen) was obtained from the Research Centre for Cultivar Testing COBORU (Cicibór Duży, Poland). All wheat samples were cleaned using mechanical dockage testers with air cyclones to remove impurities before milling. After cleaning, all samples were tempered to 15% moisture and conditioned for 24 h. After conditioning, the wheat samples’ moisture content was determined again to confirm the desired level. A second tempering event, up to 16.5% moisture, was carried out 20 min before the milling process. It was applied to toughen the skin so it would resist powdering during milling. Milling was performed using three-roller laboratory Sadkiewicz Instruments mill (Figure S1 in Supplementary Materials). Four samples of stream flour (A, B, C, D), two bran fractions and one shorts fraction were collected for each wheat cultivar (Figure S1 and Table S1 in Supplementary Materials). To diversify the ash content, some of the samples were prepared by mixing, in different proportions, flours and bran obtained from the same wheat cultivar (Table S2 in Supplementary Materials). The granulation of samples was maintained to be smaller than 160 μm.

### 2.2. Moisture and Ash Analysis

Moisture analysis was performed according to American Association of Cereal Chemists (AACC) method 44-15A [16]. The flour samples (3 g) were measured into glass weighing bottles and placed in a laboratory dryer for 3 h. The samples were dried at 105 °C to constant weight. After cooling, the samples were weighed, and the moisture contents were calculated (Table S2 in Supplementary Materials).

The ash content was determined using AACC method 08–01 [17]. The flour samples were measured into ash dishes in amounts of 3–5 g. Then samples were placed in a muffle furnace at 550 °C. They were incinerated until light gray ash or constant weight was obtained (7 h). After cooling, the samples were weighed, and the ash contents were calculated (Table S2 in Supplementary Materials).

Moisture and ash analysis was performed in triplicate. The obtained data were used to calculate mean values and standard deviations.

### 2.3. Elemental Analysis 

Elemental analysis was performed using an Elementar Vario EL Cube CHNS combustion analyzer with a thermal conductivity detector. Samples weighing 10 mg were collected from the analyzed flours in duplicate. Nitrogen, carbon, hydrogen, and sulfur content was determined, while oxygen content was calculated as the difference between the total weight and the other elements’ content.

### 2.4. Raman Spectra

Raman spectra were recorded using a Thermo Scientific iS50 Raman Module equipped with an InGaAs detector and CaF_2_ beamsplitter. Samples in the form of pellets were placed on an XYZ motorized stage. The spectra were excited using an Nd:YAG laser operating at 1064 nm, with power at the sample equal to 150 mW. Backscattered radiation was collected. Due to the samples’ inhomogeneity, the spectra were recorded from 16 different points. For each of them, interferograms were averaged over eight scans, and Happ-Genzel apodized and Fourier transformed using a zero-filling factor of 2 to yield spectra in the 200–3700 cm^−1^ range at a resolution of 8 cm^−1^. Finally, the spectra were averaged.

### 2.5. Data Analysis 

Partial least squares (PLS) models were built using Turbo Quant Analyst version 9 chemometrics software, which utilizes the nonlinear iterative partial least squares (NIPALS) algorithm [18]. A cross-validation procedure using the leave-two-out technique was performed to estimate the performance of the models. To determine the predictive abilities of the obtained models, relative standard errors of prediction were calculated for calibration and validation samples, according to the procedure described elsewhere [19]. The root means square error of cross-validation (RMSECV) was calculated to select the optimal number of PLS factors. Regression models were constructed combining MSC (multiplicative scatter correction) corrected spectra and the results of reference gravimetric and elemental analysis.

## 3. Results and Discussion

**Figure 1:**
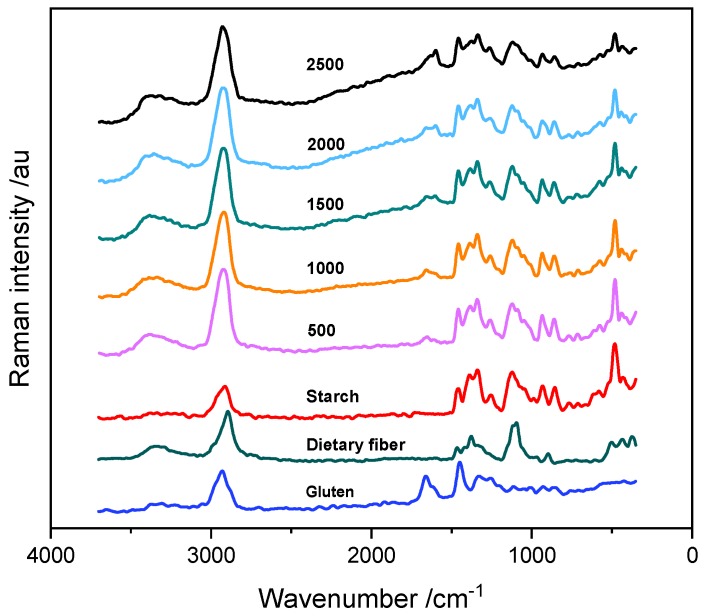
Raman spectra of various flour types and their selected components.

In Figure 1, the Raman spectra are presented for the five types of wheat flours with different ash content that were analyzed; they are labeled according to PN-A-74022:2003. The broad band with a maximum of around 3320 cm^−1^ corresponds to the O-H and N-H stretching bands. The massive band in the 2800–3000 cm^−1^ range consists of the C-H stretching vibration bands of the various flour ingredients. The bands with maxima located at 1630, 1530, and 1235 cm^−1^ are related to the I, II and III amide vibration modes. Their intensity increases with the ash content. The bands with maxima at 1460, 1385, 1339, 1128, and 1082 cm^−1^ can be assigned to different deformation modes of CH, COC, and CCC moieties in starch and dietary fiber. An intense, characteristic band of COC deformation vibration of starch molecules is observed at 479 cm^−1^ [20]. The fluorescent background is more pronounced for the samples with higher ash content. 

**Figure 2:**
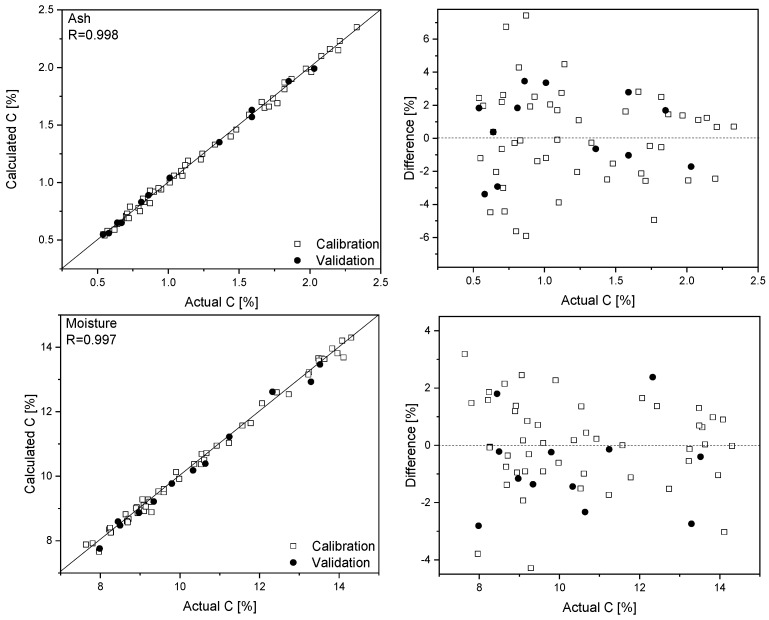
Prediction plots and regression residuals for ash (**top**) and moisture (**bottom**) quantification based on Raman spectra.

Forty-nine samples, characterized by an ash content in the 0.5–2.5% range and moisture content in the 7.6–14.3% range, were used for training purposes, while 12 were randomly selected for validation of the obtained models and six were omitted (Table S2 in Supplementary Materials). For ash modeling, 975–1790 and 2770–3580 cm^−1^ spectral ranges were applied. In the case of moisture analysis, slightly different regions were utilized (814–1770 and 2850–3696 cm^−1^). Regression coefficients plots for ash and moisture are presented in Figure S2 in Supplementary Materials. In these plots, characteristic spectral features mentioned above, can be easily identified. In the case of ash analysis, they include ν(CH), ν(C-C), and ν(C-O) vibrations together with amide I, II and III modes, and deformation motions of CH, COC, and CCC moieties. For moisture, except for ν(OH) and δ(OH) bands, contributions from different functional groups forming hydrogen bonds with water molecules can be recognized. The number of latent variables, determined from the RMSECV plots, was set to four for both analytes. The prediction plots and regression residuals for ash and moisture determination based on the Raman spectra are shown in Figure 2. The detailed model parameters are collected in Table 1. It follows from the presented results that the quantification errors of ash and moisture determination for wheat flour samples are of the order of 2%. What is important is that both analytes were determined for each sample from its Raman spectrum collected in 2 min.

The model elaborated for ash determination can be easily modified for ash content up to 5% by incorporation of the data for the whole wheat flour samples (Tables S2 and S3 and Figure S3 in Supplementary Materials).

The established amounts of carbon, hydrogen, nitrogen, oxygen and sulfur in the studied flours varied in the 40.9–42.6, 6.0–7.2, 1.9–3.0, 47.8–50.2, and 0.07–0.14% (*w*/*w*) ranges, respectively. The quantification error, expressed as the relative range, was 2–3 times higher for hydrogen quantification than for the other elements, except for sulfur. Detailed information on the results of this analysis is presented in Table S4 in Supplementary Materials.

**Figure 3:**
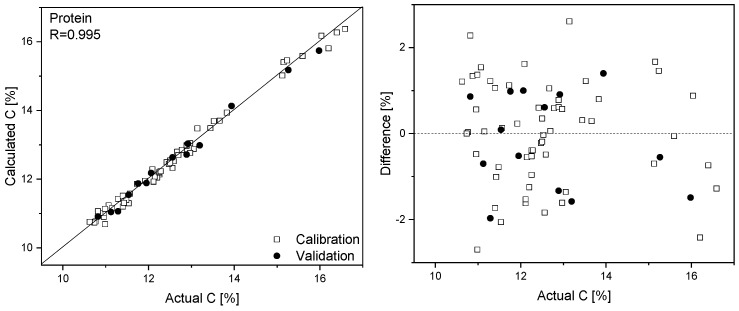
Prediction plot and regression residuals for protein quantification based on Raman spectra.

By combining spectra and the elemental analysis results, PLS calibration models that enabled the determination of N, C, S, and O content in the analyzed flours based on their Raman spectra were obtained. The parameters of these models are gathered in Table 2, while the prediction plots and residual errors are presented in Figures S4–S7 in Supplementary Materials. We were unable to construct a reliable model for hydrogen quantification. The protein amount in the analyzed flours can be determined by multiplying the nitrogen content by a factor of 5.52 [21] (Figure 3).

## 4. Conclusions

FT-Raman spectroscopy, combined with chemometric methods, appears to be an attractive tool for accurate and effective analysis of wheat flour samples. It enables quantitative determination of ash, moisture, and protein content in the analyzed samples and is much faster than the methods commonly used now.

## Figures and Tables

**Table 1:** Calibration parameters of the PLS models for ash and moisture determination.

Parameter	Ingredient	
	Ash	Moisture
R	0.998	0.997
R_cv_	0.933	0.841
RSEP_cal_	2.35	1.41
RSEP_val_	2.06	1.75

**Table 2:** Calibration parameters of the PLS models for N, C, S, and O quantification of in wheat flour.

Parameter	Element			
	Nitrogen	Carbon	Sulfur	Oxygen
R	0.995	0.995	0.974	0.979
R_cv_	0.965	0.878	0.812	0.874
RSEP_cal_	1.18	0.08	3.41	0.25
RSEP_val_	1.13	0.10	3.30	0.28
Number of factors	3	4	3	3

## Supplementary Materials

The following are available online at https://www.mdpi.com/2304-8158/9/3/280/s1, Table S1: Ash and moisture content in the products of milling; in % (*w*/*w*), Table S2: Ash and moisture content in the analyzed samples; in % (*w*/*w*), Table S3: Calibration parameters of the PLS model for ash quantification in the 0.5–5% range, Table S4: Elemental composition of the analyzed flours; in % (*w*/*w*), Figure S1 Mill flow for common wheat (Triticum aestivum) using the Sadkiewicz Laboratory mills, Figure S2: Regression coefficients plots for PLS modeling of ash, moisture and protein, Figure S3: Prediction plot and regression residuals for ash quantification in the 0.5–5% range based on Raman spectra, Figure S4: Prediction plot and regression residuals for nitrogen quantification based on Raman spectra, Figure S5: Prediction plot and regression residuals for carbon quantification based on Raman spectra, Figure S6: Prediction plot and regression residuals for sulfur quantification based on Raman spectra, Figure S7: Prediction plot and regression residuals for oxygen quantification based on Raman spectra.
