# Supplementary material for: Quantification of Ash and Moisture in Wheat Flour by Raman Spectroscopy
Source: Foods. 2020 Mar 3;9(3):280. doi: 10.3390/foods9030280 (PMC7143060; doi:10.3390/foods9030280)
Supplement: Supplementary file 1 [file foods-09-00280-s001.pdf]

Quantification of ash and moisture in wheat flour by FT-Raman spectroscopy

Tomasz Czaja [1,2], Aldona Sobota [3] and Roman Szostak [1*]

- Department of Chemistry, University of Wrocław, 14F. Joliot-Curie, 50-383 Wrocław, Poland
- Department of Food Science, University of Copenhagen, Rolighedsvej 26, 1958, Frederiksberg, Denmark
- Engineering and Cereals Technology Department, University of Life Sciences in Lublin, Skromna 8, 20-704 Lublin, Poland

Supplementary Materials

List of content:

- **Table S1** Ash and moisture content in the products of milling; in % (w/w)
- **Table S2** Ash and moisture content in the analyzed samples; in % (w/w)
- **Table S3** Calibration parameters of the PLS model for ash quantification in the 0.5-5% range
- **Table S4** Elemental composition of the analyzed samples; in % (w/w)
- **Figure S1** Mill flow for common wheat (*Triticum aestivum*) using the Sadkiewicz Laboratory mill
- **Figure S2** Regression coefficients plots for PLS modeling of ash, moisture and protein
- **Figure S3** Prediction plot and regression residuals for ash quantification in the 0.5-5% range based on FT-Raman spectra
- **Figure S4** Prediction plot and regression residuals for nitrogen quantification based on FT-Raman spectra
- **Figure S5** Prediction plot and regression residuals for carbon quantification based on FT-Raman spectra
- **Figure S6** Prediction plot and regression residuals for sulfur quantification based on FT-Raman spectra
- **Figure S7** Prediction plot and regression residuals for oxygen quantification based on FT-Raman spectra

Table S1 Ash and moisture content in the products of milling; in % (w/w)

Wheat cultivar	Ash					Moisture				
	Flour A	Flour B	Flour C	Flour D	Bran/ shorts	Flour A	Flour B	Flour C	Flour D	Bran/ shorts
Kamelia	0.68	0.56	0.79	1.09	5.14	7.80	9.14	8.43	8.07	7.01
Katoda	0.64	0.54	0.86	1.12	4.61	12.33	14.3	12.73	10.33	9.38
Monsun	0.61	0.54	0.79	1.04	5.16	14.80	13.83	8.58	12.06	12.8
Nawra	0.71	0.64	0.93	1.34	4.50	12.43	14.11	11.24	9.20	8.52
Ostka Smolicka	0.69	0.54	0.87	1.14	4.43	13.96	13.24	8.67	9.59	8.79
Raweta	0.70	0.59	0.89	1.09	4.84	13.48	11.77	10.33	8.63	7.90
Goplana	0.71	0.58	0.83	1.12	4.72	10.92	8.55	8.69	7.85	7.58
Tybalt	0.72	0.57	0.81	1.24	3.75	13.23	13.29	8.00	9.28	6.30
Fala	0.62	0,59	0.81	1.01	5.31	11.57	9.64	9.46	9.10	7.98
Kandela	0.73	0.63	0.87	1.30	4.23	13.63	13.52	11.78	9.23	10.29
Bogatka	0.74	0.66	0.87	1.10	5.33	10.12	10.64	9.16	8.91	8.54
Smuga	0.67	0.55	0.9	1.15	4.44	14.57	14.08	11.23	8.82	9.12
Pokusa	0.64	0.58	0.82	1.33	4.25	10.86	9.01	10.55	10.53	7.71
Tonacja	0.70	0.58	0.95	1.23	4.86	10.67	13.49	9.90	9.24	8.60
Skagen	0.80	0.61	1.01	1.36	5.24	10.36	13.56	9.20	9.09	8.53

Table S2 Ash and moisture content in the analyzed samples; in % (w/w)

Number	Wheat cultivar	Composition of samples	Ash	Moisture
1	Monsun	Flour B (100 %)	0.54^{x}	13.83
2	Ostka Smolicka	Flour B (100 %)	0.54^{vx}	13.24
3	Katoda	Flour B (100 %)	0.54^{xx}	14.30
4	Smuga	Flour B (100 %)	0.55^{x}	14.08
5	Tybalt	Flour B (100 %)	0.57^{vx}	13.29^{v}
6	Tonacja	Flour B (100 %)	0.58^{xx}	13.49
7	Skagen	Flour B (100 %)	0.61^{x}	13.56
8	Fala	Flour A (100 %)	0.62	11.57
9	Kandela	Flour B (100 %)	0.63^{xx}	13.52^{v}
10	Nawra	Flour B (100 %)	0.64^{x}	14.11
11	Katoda	Flour A (100 %)	0.64^{vx}	12.33^{v}
12	Bogatka	Flour B (100 %)	0.66^{x}	10.64^{v}
13	Smuga	Flour A (100 %)	0.67^{v}	14.57^{x}
14	Ostka Smolicka	Flour A (100 %)	0.69^{xv}	13.96
15	Tonacja	Flour A (100 %)	0.70^{x}	10.67
16	Raweta	Flour A (100 %)	0.70	13.48
17	Nawra	Flour A (100 %)	0.71	12.43
18	Goplana	Flour A (100 %)	0.71	10.92
19	Tybalt	Flour A (100 %)	0.72	13.23
20	Kandela	Flour A (100 %)	0.73^{x}	13.63
21	Monsun	Flour C (100 %)	0.79	8.58^{x}
22	Skagen	Flour A (100 %)	0.80^{x}	10.36
23	Fala	Flour C (100 %)	0.81^{x}	9.46
24	Tybalt	Flour C (100 %)	0.81^{vx}	8.00^{x}
25	Pokusa	Flour C (100 %)	0.82^{x}	10.55
26	Goplana	Flour C (100 %)	0.83^{x}	8.69
27	Katoda	Flour C (100 %)	0.86^{v}	12.73
28	Kandela	Flour C (100 %)	0.87	11.78
29	Ostka Smolicka	Flour C (100 %)	0.87^{x}	8.67
30	Smuga	Flour C (100 %)	0.90	11.23
31	Nawra	Flour C (100 %)	0.93^{v}	11.24^{v}
32	Tonacja	Flour C (100 %)	0.95^{x}	9.90
33	Skagen	Flour C (100 %)	1.01^{vx}	9.20
34	Fala	Flour D (100 %)	1.01^{x}	9.10
35	Monsun	Flour D (100 %)	1.04^{x}	12.06
36	Kamelia	Flour D (100 %)	1.09	13.37^{x}
37	Raweta	Flour D (100 %)	1.09^{v}	8.63
38	Bogatka	Flour D (100 %)	1.10	8.91
39	Katoda	Flour D (100 %)	1.12	10.33^{v}
40	Ostka Smolicka	Flour D (100 %)	1.14	9.59
41	Tonacja	Flour D (100 %)	1.23	9.24
42	Tybalt	Flour D (100 %)	1.24^{x}	9.28
43	Pokusa	Flour D (100 %)	1.33^{x}	10.53
44	Skagen	Flour D (100 %)	1.36^{vx}	9.09

45	Kamelia	Flour C (85 %) /bran (15%)	1.44[x]	8.22
46	Kamelia	Flour B (80 %) /bran (20 %)	1.48[x]	8.71
47	Pokusa	Flour C (80 %) /bran (20%)	1.51[x]	9.98
48	Kamelia	Flour A (80 %) /bran (20%)	1.57	7.64
49	Kandela	Flour D (90 %) /bran (10%)	1.59[v]	9.34[v]
50	Kamelia	Flour D (87.5%) /bran (12.5%)	1.59[v]	7.94
51	Bogataka	Flour A (80 %) /bran (20 %)	1.66	9.80[v]
52	Pokusa	Flour B (70 %) /bran (30 %)	1.68	8.49[v]
53	Goplana	Flour B (72.7 %) /bran (27.3 %)	1.71[v]	8.28
54	Raweta	Flour D (79.5 %) /bran (20.5 %)	1.74	8.44[v]
55	Goplana	Flour D (90 %) /bran (10 %)	1.77[x]	7.80
56	Goplana	Flour B (70.2 %) /bran (29.8 %)	1.82[x]	8.27
57	Goplana	Flour B (70 %) /bran (30 %)	1.82[x]	8.26
58	Smuga	Flour D (78.7 %) /bran (21.3 %)	1.85[v]	8.88
59	Raweta	Flour B (70 %) /bran (30 %)	1.87	10.61
60	Nawra	Flour D (80 %) /bran (20 %)	1.97[v]	9.06
61	Fala	Flour B (70 %) /bran (30 %)	2.01	9.14
62	Skagen	Flour D (82.5 %) /bran (17.5 %)	2.03	8.99
63	Pokusa	Flour A (60 %) /bran (40 %)	2.08[v]	9.60[x]
64	Ostka Smolicka	Flour D (70.5 %) /bran (29.5 %)	2.11[v]	9.34
65	Smuga	Flour D (70 %) /bran (30 %)	2.14[x]	8.91[x]
66	Bogatka	Flour C (70 %) /bran (30 %)	2.21	8.97[v]
67	Tybalt	Flour D (50 %) /bran (50 %)	2.24	7.17[v]
68	Monsun	Flour A (47 %) /bran (53 %)	3.02[x]	12.90[x]
69	Smuga	Flour A (31.5 %) /bran (68.5 %)	3.22[xv]	10.84[x]
70	Monsun	Flour D (46 %)/bran (54 %)	3.26[x]	12.45[x]
71	Monsun	Flour A (40 %)/bran (60 %)	3.34[x]	12.72[x]
72	Kandela	Bran (100 %)	4.23[x]	10.29[x]
73	Ostka Smolicka	Bran (100 %)	4.43[xv]	8.79[x]
74	Smuga	Bran (100 %)	4.44[x]	9.12[x]
75	Katoda	Bran (100 %)	4.61[x]	9.38[x]
76	Tonacja	Bran (100 %)	4.86[x]	8.60[x]
77	Skagen	Bran (100 %)	5.24[x]	8.53[x]

[v] – validation sample; [x] – ignored sample; red color – ash in the 0.5-2.2% range; blue color – ash in the 0.5-5.2% range

Table S3 Calibration parameters of the PLS model for ash quantification in the 0.5-5.2% range

**Parameter**	
R	0.998
R_{cv}	0.953
$RSEP_{cal}$	3.54
$RSEP_{val}$	3.59

Table S4 Elemental composition of the analyzed flours; in % (w/w)

Number	Nitrogen	Carbon	Sulfur	Oxygen
1	2.015[v]	41.400	0.075	49.579[x]
2	1.960[v]	40.930	0.097	50.223
3	2.020	41.175[v]	0.073[v]	49.665
4	1.970	41.440	0.083[v]	49.519
5	1.945	41.015	0.097	50.160
6	1.990	41.135	0.073	49.750[v]
7	1.925	41.065[v]	0.107	50.154
8	1.985	41.130	0.085	49.707
9	1.960	41.545	0.083	49.447
10	2.045[v]	41.265[v]	0.078	49.560
11	2.065	41.565	0.072	49.632[v]
12	1.985	41.150	0.083	49.634
13	2.230[x]	41.245	0.082	49.363
14	2.225	41.615	0.083	49.157
15	2.090[v]	41.230	0.087	50.574[x]
16	2.190	41.135[x]	0.100	49.793
17	2.005	41.410	0.051[x]	50.306
18	2.080	41.160	0.084	49.922
19	2.070	41.620	0.078	49.288
20	1.990	41.665	0.086	49.303
21	1.950	41.180	0.083	49.875[v]
22	2.040[x]	41.315	0.089[v]	49.416
23	2.090	41.275	0.080	49.396
24	2.045	41.265	0.091[v]	49.486
25	2.065	41.270	0.089	49.400
26	2.130[v]	41.325	0.094	49.364
27	2.195	41.725	0.083[v]	49.068
28	2.220	41.240	0.093	49.276
29	2.095	41.330	0.100	49.662
30	2.185[v]	41.295	0.092	49.324
31	2.220	41.345[v]	0.093[v]	49.480[v]
32	2.280	41.320	0.097	49.174[v]
33	2.195	41.375[v]	0.094	49.429[v]
34	2.220	41.295[v]	0.109[x]	49.298
35	2.160	41.590	0.072	49.349
36	2.275[v]	41.340	0.085	49.235
37	2.125	41.450[v]	0.087	49.888
38	2.165[v]	41.495	0.094[v]	49.180
39	2.200	41.455	0.093	49.344[v]
40	2.265	41.471	0.087	49.000
41	2.210	41.445	0.095	49.143
42	2.235[x]	41.510	0.100[v]	48.986
43	2.275	41.375	0.097[v]	49.456
44	2.270	41.470	0.108	49.057

45	2.295	41.735[v]	0.090	48.814[x]
46	2.335[v]	41.640[v]	0.138[x]	49.202[v]
47	2.250	41.560	0.097	48.981[v]
48	2.300	41.560	0.086	49.287
49	2.365	41.490[x]	0.093	49.102[v]
50	2.350	41.630[v]	0.097	48.854[v]
51	2.315	41.590	0.097	48.998
52	2.340[v]	41.675[v]	0.097	48.849
53	2.260	41.710[v]	0.097	49.172
54	2.335	41.660	0.101	48.847
55	2.265	41.575	0.100	49.027
56	2.345[x]	41.650	0.105	48.773
57	2.220[x]	41.760	0.113[v]	49.855[x]
58	2.380	41.340[x]	0.104	49.539
59	2.390[v]	41.755	0.109	48.735
60	2.335	41.545	0.095	49.128
61	2.350	41.660	0.104	48.882
62	2.435	41.825	0.104[v]	48.697[v]
63	2.475	41.845	0.101	48.972
64	2.260	41.605	0.136	48.973
65	2.450[x]	42.140 [x]	0.108[v]	48.250
66	2.525	42.055	0.111	48.261
67	2.505[v]	42.040 [x]	0.112	48.343
68	2.825	42.165	0.113	47.849
69	2.740	42.110[v]	0.098	48.076
70	2.765[v]	42.565	0.110[v]	47.620
71	2.760	42.410	0.101[x]	47.869
72	2.895[v]	42.325[v]	0.128	48.158[v]
73	2.970	42.230	0.133[v]	47.834[v]
74	2.745	42.355	0.135	47.659[x]
75	2.935	42.500	0.135	47.331[x]
76	3.005	42.215	0.248[x]	48.213
77	2.905	42.275[x]	0.176[x]	47.662

[v] – validation sample; [x] – ignored sample;

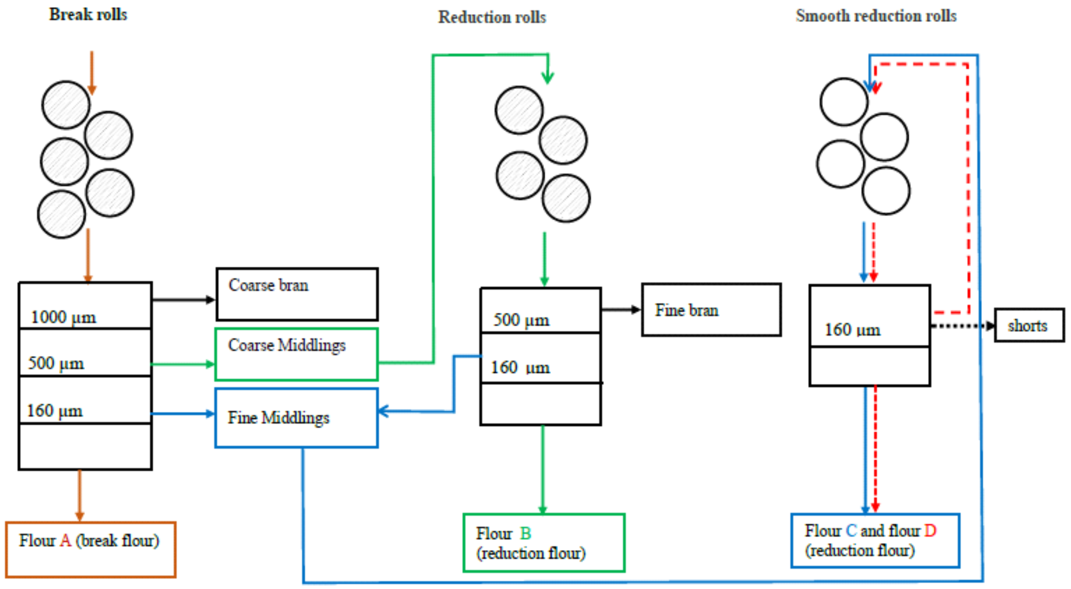

Figure S1 Mill flow for common wheat (*Triticum aestivum*) using the Sadkiewicz Laboratory mills

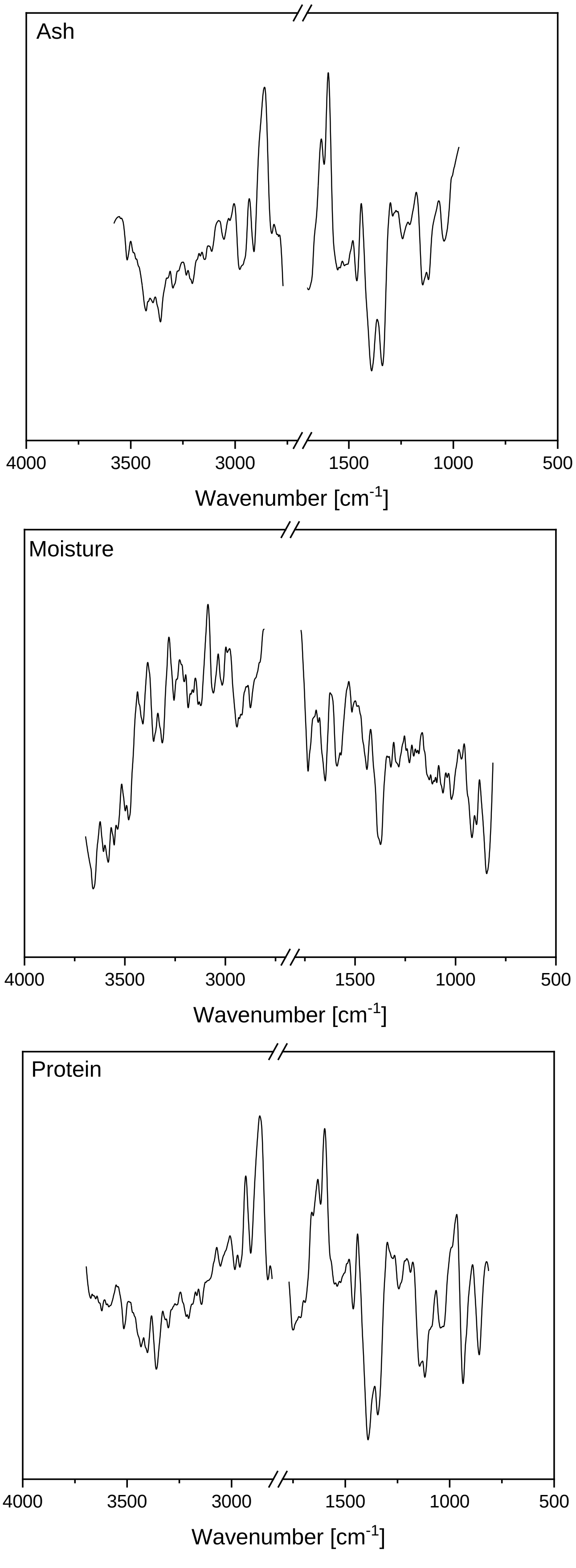

Figure S2 Regression coefficients plots for PLS modeling of ash, moisture and protein

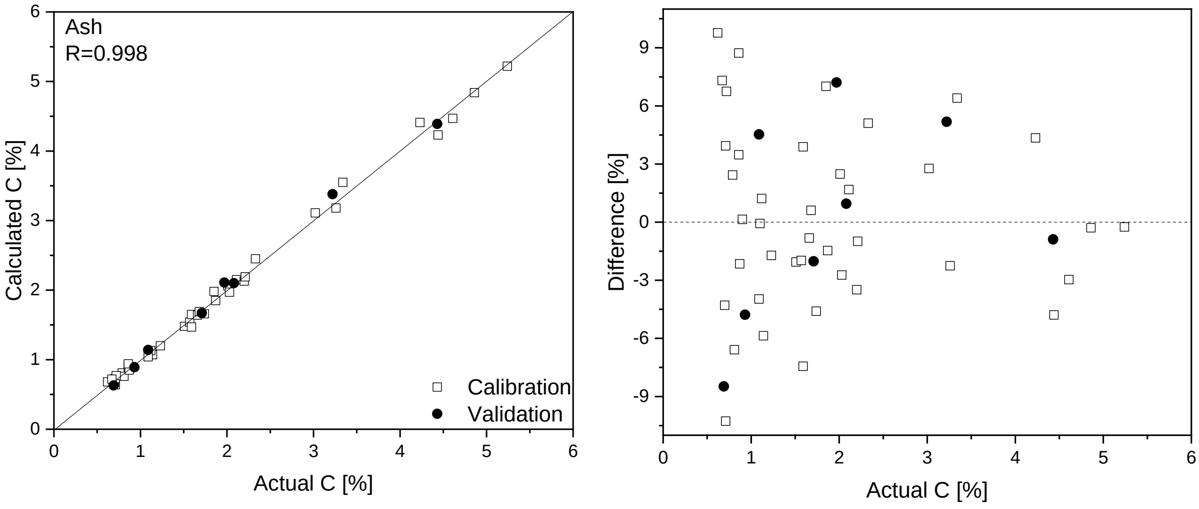

Figure S3 Prediction plot and regression residuals for ash quantification in the 0.5-5% range based on FT-Raman spectra

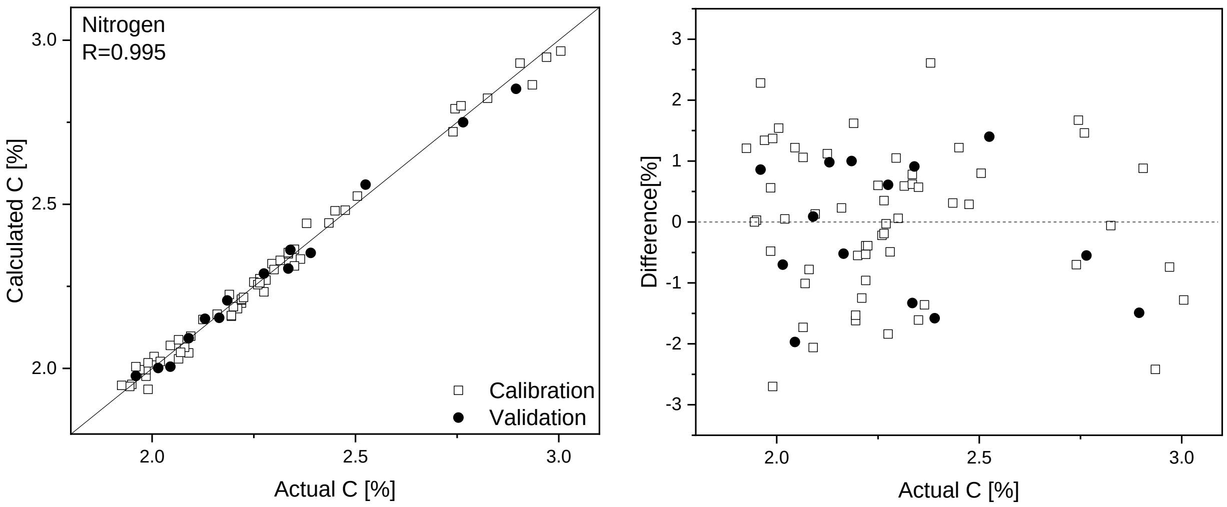

Figure S4 Prediction plot and regression residuals for nitrogen quantification based on FT-Raman spectra

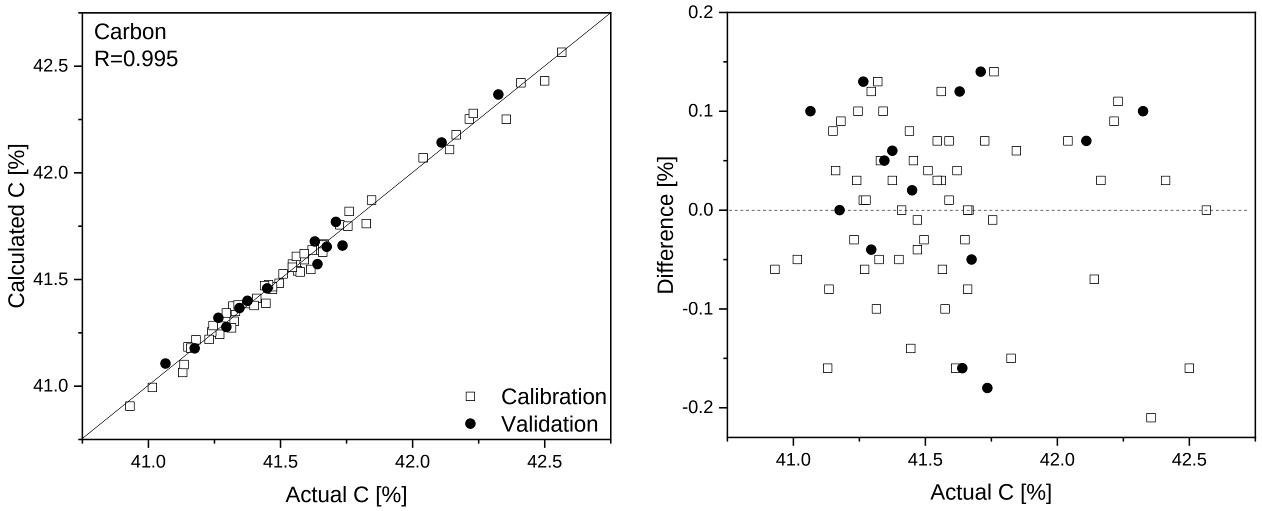

Figure S5 Prediction plot and regression residuals for carbon quantification based on FT-Raman spectra

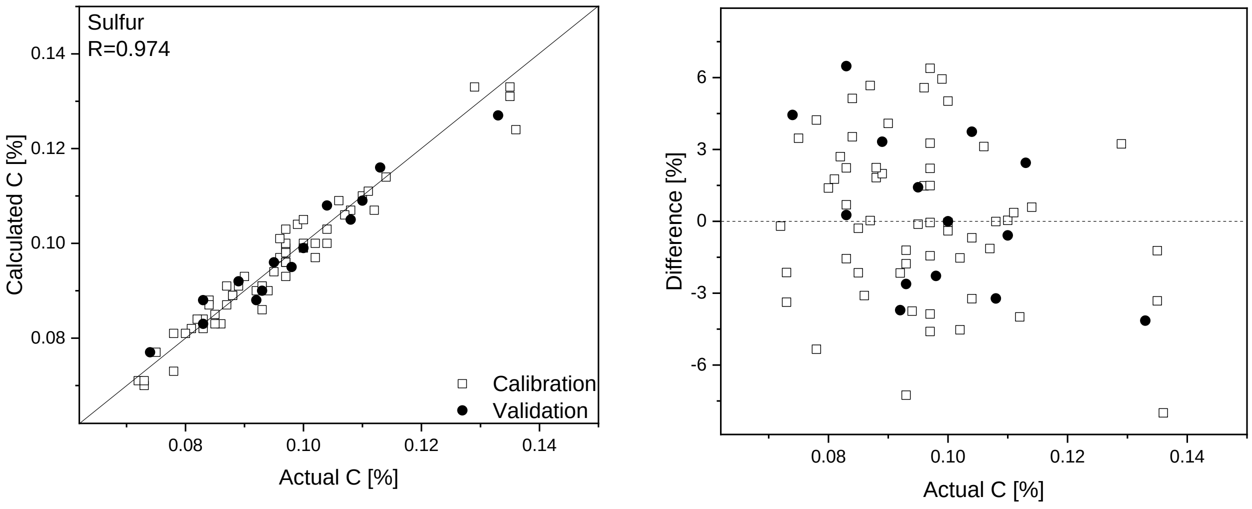

Figure S6 Prediction plot and regression residuals for sulfur quantification based on FT-Raman spectra

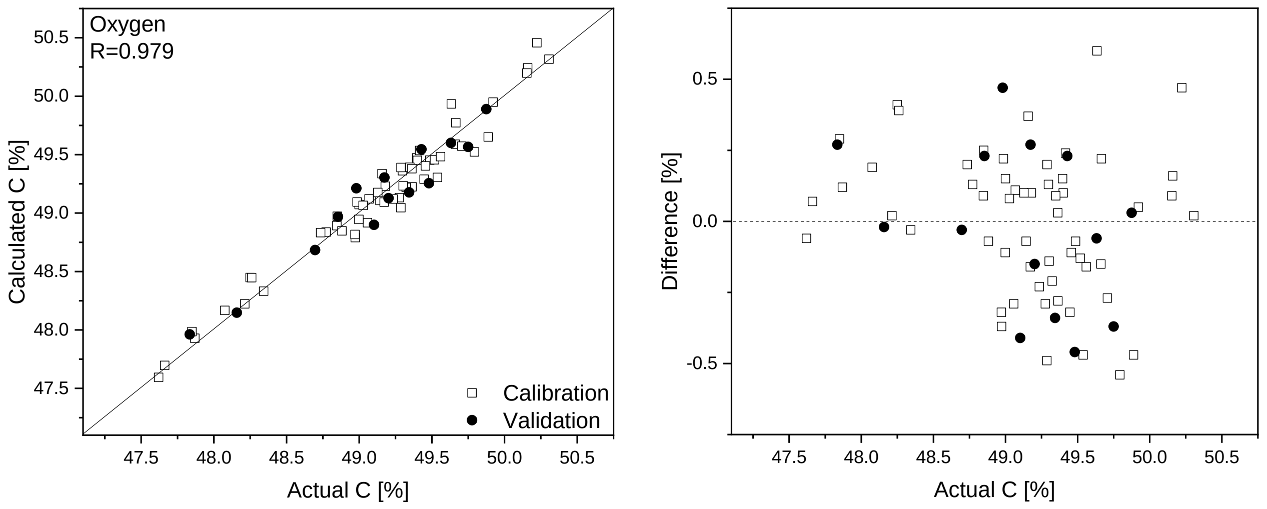

Figure S7 Prediction plot and regression residuals for oxygen quantification based on FT-Raman spectra
